# Engineering Strategies to Decode and Enhance the Genomes of Coral Symbionts

**DOI:** 10.3389/fmicb.2017.01220

**Published:** 2017-06-30

**Authors:** Rachel A. Levin, Christian R. Voolstra, Shobhit Agrawal, Peter D. Steinberg, David J. Suggett, Madeleine J. H. van Oppen

**Affiliations:** ^1^Centre for Marine Bio-Innovation, The University of New South Wales, SydneyNSW, Australia; ^2^School of Biological, Earth and Environmental Sciences, The University of New South Wales, SydneyNSW, Australia; ^3^Climate Change Cluster, University of Technology Sydney, UltimoNSW, Australia; ^4^Red Sea Research Center, Division of Biological and Environmental Science and Engineering (BESE), King Abdullah University of Science and Technology (KAUST),Thuwal, Saudi Arabia; ^5^Sydney Institute of Marine Science, MosmanNSW, Australia; ^6^Australian Institute of Marine Science, TownsvilleQLD, Australia; ^7^School of BioSciences, The University of Melbourne, ParkvilleVIC, Australia

**Keywords:** synthetic biology, genetic engineering, dinoflagellate, *Symbiodinium*, zooxanthellae, coral bleaching

## Abstract

Elevated sea surface temperatures from a severe and prolonged El Niño event (2014–2016) fueled by climate change have resulted in mass coral bleaching (loss of dinoflagellate photosymbionts, *Symbiodinium* spp., from coral tissues) and subsequent coral mortality, devastating reefs worldwide. Genetic variation within and between *Symbiodinium* species strongly influences the bleaching tolerance of corals, thus recent papers have called for genetic engineering of *Symbiodinium* to elucidate the genetic basis of bleaching-relevant *Symbiodinium* traits. However, while *Symbiodinium* has been intensively studied for over 50 years, genetic transformation of *Symbiodinium* has seen little success likely due to the large evolutionary divergence between *Symbiodinium* and other model eukaryotes rendering standard transformation systems incompatible. Here, we integrate the growing wealth of *Symbiodinium* next-generation sequencing data to design tailored genetic engineering strategies. Specifically, we develop a testable expression construct model that incorporates endogenous *Symbiodinium* promoters, terminators, and genes of interest, as well as an internal ribosomal entry site from a *Symbiodinium* virus. Furthermore, we assess the potential for CRISPR/Cas9 genome editing through new analyses of the three currently available *Symbiodinium* genomes. Finally, we discuss how genetic engineering could be applied to enhance the stress tolerance of *Symbiodinium*, and in turn, coral reefs.

## Introduction

Photosynthetic dinoflagellates are critical primary producers in the aquatic environment, yet, their functional genomics are largely unexplored (Leggat et al., 2011; Murray et al., 2016). *Symbiodinium* is considered one of the most important dinoflagellate genera given its role as the essential photosymbiont of many tropical reef invertebrates, notably reef-building corals (Trench and Blank, 1987). Provision of photosynthetically derived metabolites from *Symbiodinium* to the coral host drives coral calcification and growth that forms the foundation of coral reef ecosystems (Muscatine and Porter, 1977; Muscatine, 1990; Kirk and Weis, 2016). Thermal and light stress cause photosynthetic dysfunction of *Symbiodinium* and increased leakage of harmful reactive oxygen species from their cells, a process considered largely responsible for the dissociation of *Symbiodinium* from corals characterized as “coral bleaching” (Warner et al., 1999; Suggett et al., 2008; Weis, 2008; Levin et al., 2016). *Symbiodinium* has therefore become established as a major focus for research globally, and in effect, a model genus for dinoflagellates.

Dinoflagellates evolved an estimated 520 million years ago (Moldowan and Talyzina, 1998) and exhibit substantial evolutionary divergence from model eukaryotic organisms including other microalgae such as *Chlamydomonas* and diatoms (Shoguchi et al., 2013). Consequently, dinoflagellates possess unusual biological features that have hindered research progress, such as some of the largest known nuclear genomes (1.5–112 Gbp, typically exceeding the size of the human haploid genome), permanently condensed liquid-crystalline chromosomes, trans-splicing of polycistronic mRNAs, and plastid genomes that are divided up into minicircles (Shoguchi et al., 2013; Zhang et al., 2013; Lin et al., 2015; Murray et al., 2016). The *Symbiodinium* genus evolved an estimated 50 million years ago and is highly diverse, containing nine major evolutionary lineages or “clades” (A–I; Coffroth and Santos, 2005; Pochon et al., 2006; Pochon and Gates, 2010) with hundreds of genetically distinct “types/sub-clades” considered to be different species^1^ (Tonk et al., 2013). Genetic factors that promote differences in stress tolerance between *Symbiodinium* variants (both inter- and intra-specific) strongly influence coral gene expression and bleaching susceptibility (Berkelmans and van Oppen, 2006; DeSalvo et al., 2010; Yuyama et al., 2012; Levin et al., 2016). However, the capacity to fully explore *Symbiodinium* genetics is currently restricted by a lack of genetic engineering capability. Genetic engineering has been central to the study of gene function and phenotypic enhancement in organisms ranging from microbes to mammals and a key platform for socioeconomic industries and biotechnologies; yet only two cases of transgene expression in *Symbiodinium* have ever been validated (ten Lohuis and Miller, 1998; Ortiz-Matamoros et al., 2015a).

In 1998, a type A1 strain was transformed at very low efficiencies using silicon carbide whiskers with plasmids encoding expression constructs with plant, plant-viral, and agrobacterial promoters (nos, CaMV 35S, and p1′2′) to drive transcription of antibiotic resistance genes (*nptII* and *hptII*) and a reporter gene (*GUS*) (ten Lohuis and Miller, 1998); however, these results have yet to be reproduced. It was not until 2015 that another case of transgene expression in *Symbiodinium* was reported (Ortiz-Matamoros et al., 2015b). Plasmids encoding expression constructs with plant and plant-viral promoters (nos and double CaMV 35S) to drive transcription of a herbicide resistance gene (*bar*) and a reporter gene (*GFP*) were introduced to type A1, B1, and F1 strains using glass beads. Whilst cells transiently exhibited improved herbicide resistance and suggestive *GFP* signal, transformations were not validated through DNA, RNA, or protein analysis (Ortiz-Matamoros et al., 2015b). Further transformation of these strains was attempted using *Agrobacterium* carrying plasmids with the same expression constructs, but the transformants were transient and unable to divide (Ortiz-Matamoros et al., 2015a). Of these studies, none attempted manipulation of ecologically relevant genes thereby limiting new insight gained into *Symbiodinium* biology.

**FIGURE 1:**
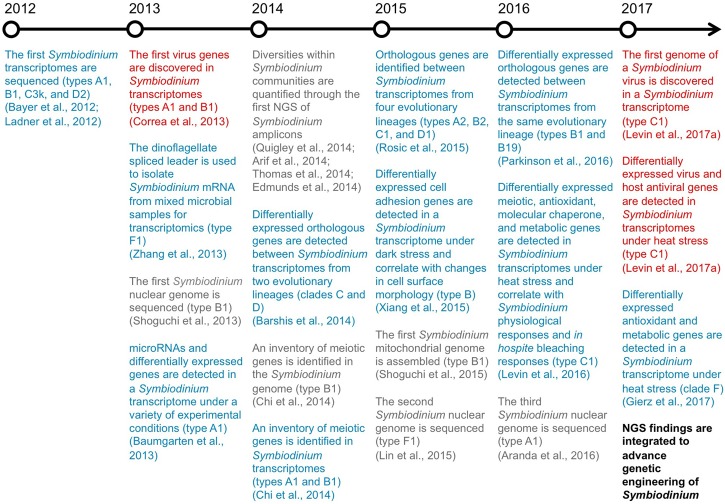
Breakthroughs in NGS of *Symbiodinium*. A timeline highlighting the key genomic (gray), transcriptomic (blue), and virus RNA (red) findings from recent NGS studies of *Symbiodinium*.

Therefore, in an attempt to overcome the bottleneck that has become established in transforming *Symbiodinium* (and other dinoflagellates), we recommend a new approach that capitalizes on the recent surge in “omics” breakthroughs (**Figure 1**). By evaluating the rapidly increasing supply of next-generation sequencing (NGS) data, we propose a genetic engineering framework for *Symbiodinium* that may markedly advance our understanding of these important dinoflagellates. Furthermore, genetic manipulation of *Symbiodinium* in order to reduce coral bleaching has been hypothesized as a strategy to facilitate coral management as reefs continue to rapidly deteriorate under climate change (van Oppen et al., 2017). Combatting the impacts of climate change and conserving marine organisms are both key goals for sustainable development set forth by the United Nations^2^. Thus, we believe genetic engineering of *Symbiodinium* may open a novel avenue to achieve these goals by protecting corals from climate change.

## Tailoring a Genetic Engineering Framework for *Symbiodinium*

Fundamental components of *Symbiodinium* biology have recently been uncovered through a boom in NGS (**Figure 1**), particularly the assembly of the first *Symbiodinium* genomes and transcriptomes, direct correlation between *Symbiodinium* transcriptional and physiological states, and discovery of genes from viruses actively infecting *Symbiodinium* cells. Furthermore, NGS of *Symbiodinium* has revealed genetic elements that may allow for transformation of *Symbiodinium*. In the following sections, we detail how unique *Symbiodinium* promoters, specific *Symbiodinium* genes underpinning important phenotypes, and a viral internal ribosomal entry site recognized by *Symbiodinium* ribosomes could be integrated to build expression constructs for *Symbiodinium*.

## Transcriptional Promoters and Terminators

Currently, dinoflagellate nuclear genome assemblies are all from the genus *Symbiodinium* (types A1, B1, and F1; Shoguchi et al., 2013; Lin et al., 2015; Aranda et al., 2016), emphasizing the importance of *Symbiodinium* to dinoflagellate research. The assemblies have revealed the immense size of *Symbiodinium* genomes with 36,850–49,109 genes, unidirectional gene orientation, prevalent gene tandem arrays, microRNAs along with putative gene targets, and unique promoter architecture (Shoguchi et al., 2013; Lin et al., 2015; Aranda et al., 2016). Rather than the traditional TATA-box of eukaryotic promoters, *Symbiodinium* promoters appear to have a TTTT-box that is followed by a unique transcription start site (YYANWYY), branch point (YTNAY), and acceptor for the dinoflagellate spliced leader (AG) (Lin et al., 2015). Additionally, instead of the typical eukaryotic polyadenylation signal AAUAAA, dinoflagellate terminators use AAAAG/C (Bachvaroff and Place, 2008). Hence, utilization of endogenous *Symbiodinium* promoters and terminators (as opposed to promoters and terminators from other organisms) would likely improve expression and stability of transgenes introduced into *Symbiodinium*. By chance, the CAMV 35S (plant-viral) promoter happens to contain all of the described *Symbiodinium* promoter elements, and the CAMV 35S (plant-viral) and nos (plant) terminators both contain the dinoflagellate polyadenylation signal; this may have contributed to their ability to drive transgene expression in *Symbiodinium* previously (ten Lohuis and Miller, 1998; Ortiz-Matamoros et al., 2015a).

**FIGURE 2:**
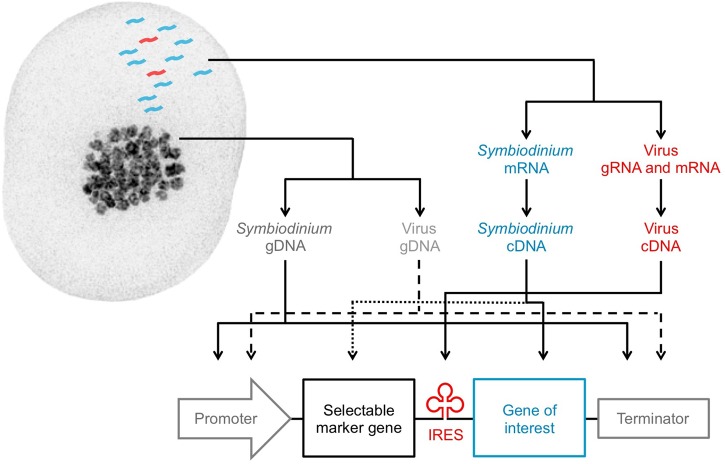
Design of a tailored expression construct for *Symbiodinium*. Genetic elements that can be isolated from *Symbiodinium* cells: *Symbiodinium* genomic DNA (dark gray), *Symbiodinium* messenger RNA (blue), resident virus genomic DNA (light gray), resident virus genomic or messenger RNA (red). Solid lines (identified elements) and dashed or dotted lines (unidentified elements) are used to arrange the elements into a *Symbiodinium* expression construct. The pictured *Symbiodinium* cell (type C1) was stained with DAPI for imaging on a DeltaVision OMX Blaze microscope (excitation/emission: 405 nm/419–465 nm).

Recent transcriptomic studies have identified highly expressed *Symbiodinium* nuclear genes that can be genome-mapped to uncover strong, endogenous promoters and their corresponding terminators. These promoters and terminators can be isolated from purified genomic DNA (gDNA) through PCR and incorporated into custom DNA expression constructs for *Symbiodinium* (**Figure 2**). Among the most highly expressed transcripts in *Symbiodinium* transcriptomes are genes for peridinin-chlorophyll *a*-binding protein, caroteno-chlorophyll *a*-*c*-binding protein, major basic nuclear protein 2, dinoflagellate viral nucleoprotein, and glyceraldehyde-3-phosphate dehydrogenase (Baumgarten et al., 2013; Levin et al., 2016; Parkinson et al., 2016); though all are multi-copy genes (Shoguchi et al., 2013; Lin et al., 2015; Aranda et al., 2016). Ideally, highly expressed nuclear genes chosen for promoter selection should not have high copy numbers, as their expression levels may largely be due to prevalence in the genome rather than strong promoters. Constitutively expressed nuclear genes are also desirable for selection of promoters that drive consistent transcription regardless of experimental conditions, and thus, drive reliable transgene expression.

To illustrate this approach of *Symbiodinium* promoter selection, we examined NGS data from a type A1 *Symbiodinium* strain for which the nuclear genome has been recently sequenced (Aranda et al., 2016) and the transcriptional responses to various conditions (temperatures, ionic stress, dark stress, and contrasting circadian rhythm time points) have been determined (Baumgarten et al., 2013). Locus 144 and Locus 1768 in the type A1 transcriptome, a subunit of a large neutral amino acids transporter and a putative ATP-binding cassette transporter gene, both show high expression across all conditions (average expression in the top 2% of all genes; Baumgarten et al., 2013) and map tightly to the type A1 genome scaffolds 710 and 484, respectively. No significant open reading frames are found >5 kb up- or down-stream of either gene, confirming that they are not part of tandem arrays. For each gene, all *Symbiodinium* promoter elements are within 1 kb of the start codon, and the dinoflagellate polyadenylation signal is found ∼300 bp after the stop codon. These promoter and terminator regions could therefore be isolated and utilized to drive high and consistent expression of transgenes in a *Symbiodinium* expression construct.

## Genes of Interest

Recent transcriptomic studies have been fundamental in the discovery of *Symbiodinium* nuclear genes that underpin phenotypic traits, such as those related to cell adhesion (e.g., *GspB*, *Svep1*, *Slap1*; Xiang et al., 2015), sexual reproduction (e.g., *Msh4*, *Msh5*, *Spo11-2*; Chi et al., 2014; Levin et al., 2016; Gierz et al., 2017), antiviral response (e.g., *Birc3*, *Ns1bp*, *Ifih1*; Levin et al., 2017a), and antioxidant activity/thermal tolerance (e.g., *Fe-sod*, *Mn-sod*, *Pxrd*, *Hsp70*; Levin et al., 2016; Gierz et al., 2017). *Symbiodinium* antioxidant genes are of particular interests because of their potential role in defining bleaching susceptibility of the coral host (Krueger et al., 2015; Levin et al., 2016). For instance, iron-type superoxide dismutase (*Fe-sod*) genes are believed to minimize thermally induced oxidative damage to photosynthetic apparatuses and leakage of harmful reactive oxygen species from type C1 *Symbiodinium* cells—determinants of coral bleaching (Weis, 2008); however, these genes are not expressed at detectable levels in all *Symbiodinium* variants (Krueger et al., 2015; Levin et al., 2016). A *Fe-sod* gene could therefore be inserted after a strong *Symbiodinium* promoter in an expression construct to drive its over-expression for evaluation of its phenotypic influence on *Symbiodinium*. Endogenous genes of interest should be isolated through PCR of complementary DNA (cDNA) reverse transcribed from purified mRNA, since gDNA introns may prevent proper expression in constructs (**Figure 2**).

Expression of exogenous genes of interest in *Symbiodinium* could also greatly advance investigations of ecological processes central to coral reef health. For instance, documenting competition between *Symbiodinium* types, transmission and acquisition of *Symbiodinium* types by the coral host, and shuffling of *Symbiodinium* types within host tissues (Toller et al., 2001; van Oppen et al., 2001; Little et al., 2004; Berkelmans and van Oppen, 2006; Byler et al., 2013; Boulotte et al., 2016) is currently reliant upon sequencing since it is not possible to visually differentiate many types. As a result, studies have been restricted to low temporal and spatial resolution relative to real-time imaging. Instead, the ability to color-code *Symbiodinium* types through genetic transformation with various fluorescent proteins could illuminate these phenomena by enabling real-time imaging for visually differentiating types. Additionally, tagging endogenous genes of interest through fluorescent protein fusions would permit imaging of protein localization within *Symbiodinium* cells and potential protein secretion out of *Symbiodinium* cells (Xiang et al., 2015). When selecting appropriate fluorescent proteins, it will be imperative to consider the extreme autofluorescence of *Symbiodinium* (Shaner et al., 2005); for example, venus (excitation/emission: 515/528 nm), tdTomato (excitation/emission: 554/581 nm), and mCherry (excitation/emission: 587/610 nm) are promising candidates as their fluorescence properties are off-peak of the *Symbiodinium* excitation and emission spectra (Hennige et al., 2009; Jiang et al., 2012). Finally, codon optimization may be necessary for optimal exogenous gene expression in *Symbiodinium* since codon usage of *Symbiodinium* genes can be divergent from foreign genes (Levin et al., 2017a) and even between *Symbiodinium* nuclear and minicircle genes (Bayer et al., 2012).

## Selectable Marker Genes

Although antibiotics have previously been used to select transformed *Symbiodinium* (ten Lohuis and Miller, 1998; Ortiz-Matamoros et al., 2015a), their use is problematic for two main reasons. Firstly, eliminating wild-type *Symbiodinium* in culture requires high concentrations of antibiotics (e.g., 3 mg/ml of G418 or hygromycin; ten Lohuis and Miller, 1998), making experimentation and long-term maintenance of transformed cell lines extremely costly. It is also important to note that natural antibiotic resistances are not uniform across all strains (Supplementary Table 1), so dosage curves are necessary before conducting transformation trials. Secondly, dinoflagellates including *Symbiodinium* require symbiotic bacteria to grow optimally (Alavi et al., 2001; Croft et al., 2005; Miller and Belas, 2006; Ritchie, 2012). Since eukaryotic antibiotics can also be toxic to prokaryotes (Gonzalez et al., 1978; Colanduoni and Villafranca, 1986; Pline et al., 2001; Vicens and Westhof, 2003), bacterial communities in *Symbiodinium* cultures are removed during antibiotic selection.

To preserve symbiotic bacteria, alternatives to antibiotic selection markers should be considered, such as genes that provide growth advantages under specific conditions by increasing pathogen resistance, increasing thermal tolerance, or allowing for utilization of non-metabolized carbohydrates (Breyer et al., 2014). The precise functions of these alternative marker genes (e.g., phosphomannose isomerase) are well defined and shown to be applicable to many photosynthetic species (Stoykova and Stoeva-Popova, 2011), though their compatibilities with dinoflagellates are unknown. Discovery of endogenous selectable markers should therefore also be pursued. Recent *Symbiodinium* transcriptomic studies have uncovered genes involved in selection-relevant phenotypes like photosynthetic ability at unique light regimes (Parkinson et al., 2016) or tolerance to increased temperature regimes (Levin et al., 2016). These *Symbiodinium* genes could first be expressed in more easily transformed microalgae like *Chlamydomonas* and diatoms to gauge the potential for their up-regulation to grant a significant selectable advantage under specific conditions.

## Viral Elements

Viral promoters and terminators, internal ribosome entry sites (IRES), and 2A peptides are staple regulatory elements incorporated in expression constructs since they have evolved to be recognized by eukaryotic machinery for efficient and stable foreign gene expression (Benfey and Chua, 1990; Martínez-Salas, 1999; Levin et al., 2014). *Symbiodinium* transcriptomics have led to the discovery of genes, as well as an entire RNA genome, from novel eukaryotic viruses that infect *Symbiodinium* (Correa et al., 2013; Levin et al., 2017a). A putative viral IRES, which allows cap-independent translation to produce separate proteins from one mRNA transcript, was found between the two open reading frames in the RNA genome of the +ssRNA virus infecting type C1 *Symbiodinium* (GenBank accession: KX538960 and KX787934; Levin et al., 2017a). The +ssRNA virus transcripts were extremely abundant in a type C1 *Symbiodinium* transcriptome (Levin et al., 2017a), and such rampant +ssRNA virus replication indicates that *Symbiodinium* ribosomes have high affinity to this IRES.

IRES sequences enable the creation of polycistronic constructs transcriptionally controlled by a single promoter (Martínez-Salas, 1999). By permitting simultaneous expression of two independent proteins from one mRNA, a bicistronic construct can achieve long-term expression of a gene of interest because the gene of interest is transcriptionally fused to the selectable marker gene (Gurtu et al., 1996; **Figure 2**). Conversely, in monocistronic constructs, the selectable marker gene often maintains expression, while the gene of interest becomes transcriptionally repressed over time if it does not increase fitness of the cell (Allera-Moreau et al., 2007). Therefore, the IRES from the *Symbiodinium* +ssRNA virus is a valuable viral element that is recognized by *Symbiodinium* ribosomes and may improve the stability of transgene expression in *Symbiodinium.* Moving forward, NGS data of viruses in *Symbiodinium* cultures (Weynberg et al., 2017) and the coral holobiont (Weynberg et al., 2014; Correa et al., 2016) should be mined for promoter, terminator, and other regulatory elements from *Symbiodinium* viruses, given the proven benefits of viral elements to genetic engineering. Once assembled, the *Symbiodinium* expression construct (**Figure 2**) can be combined with the backbone of a standard cloning plasmid; added into an artificial, replicating minicircle (Nehlsen et al., 2006; Karas et al., 2015); or serve as a repair template for CRISPR/Cas9 genome editing (Cong et al., 2013).

## Crispr/Cas9 Genome Editing and *Symbiodinium*

Within the past 5 years, CRISPR/Cas9 has revolutionized genome editing by allowing precise changes to be made to target sites in the genome (Cong et al., 2013; Baek et al., 2016; Nymark et al., 2016). In short, a single guide RNA (sgRNA) is designed to recruit the Cas9 endonuclease protein and to match a specific, desired target site in the genome that must be immediately followed by a protospacer adjacent motif (PAM) sequence (5′-NGG-3′). Once complexed with Cas9, the sgRNA guides Cas9 to the target genome site. Cas9 then interacts with the PAM sequence and creates a double-strand break in the target site. The cell can either repair the double stranded break through non-homologous end joining (NHEJ) or homology-directed repair (HDR) (Ran et al., 2013). NHEJ genome editing arises from introduction of a random mutation/insertion/deletion when the broken ends of DNA are directly ligated, which can cause the target gene to be knocked out (i.e., non-functional). Gene knockout provides insight into the role and criticality of a gene by assessing the effect of its absence. Alternatively, HDR genome editing uses a repair template flanked by 5′ and 3′ homologous arm sequences that match the up- and down-stream regions of the double-strand break. The repair template can be designed for gene knockout, introduction of a specific mutation/insertion/deletion, or genomic integration of a transgene(s)/entire expression construct (Ran et al., 2013).

*Symbiodinium* exhibits an asexual haploid vegetative stage (Santos and Coffroth, 2003) with sister chromatids developing in S-phase of the cell cycle (Watrin and Legagneux, 2003), but HDR has yet to be directly observed in *Symbiodinium*. Therefore, CRISPR/Cas9 genome editing of *Symbiodinium* may be restricted to NHEJ. *Ku70*, *Ku80*, and DNA ligase IV (genes central to NHEJ; Chu et al., 2015) are all expressed in *Symbiodinium* transcriptomes (Levin et al., 2016). That said, some evidence does suggest *Symbiodinium* can enter a transient sexual diploid stage (Chi et al., 2014; Wilkinson et al., 2015; Levin et al., 2016), which has been documented in other dinoflagellates (Figueroa et al., 2015). In yeast, ploidy shifts the dominant double-stranded break repair mechanism—diploid cells favor HDR, while haploid cells favor NHEJ (Lee et al., 1999). Moreover, genes specific to meiosis, a process during which HDR occurs (Thacker and Keeney, 2016), have been found in *Symbiodinium* genomes and transcriptomes (Chi et al., 2014; Lin et al., 2015; Rosic et al., 2015; Levin et al., 2016). *Msh4*, *Msh5*, and *Spo11-2* are all highly up-regulated at elevated temperatures (Levin et al., 2016), suggesting that HDR pathways in *Symbiodinium* are activated. *Brca2*, a gene that controls HDR (Holloman, 2011), is likewise up-regulated in heat stressed *Symbiodinium* (SM population: TR74441| c0_g1; MI population: TR63986| c0_g1; Levin et al., 2016). Hence, the potential for genomic integration of transgenes through HDR may improve if *Symbiodinium* are pre-stressed. HDR in *Symbiodinium* may also be increased by suppression of *Ku70*, *Ku80*, or DNA ligase IV (Chu et al., 2015).

The permanently condensed chromosomes of *Symbiodinium* could present an obstacle for CRISPR/Cas9 genome editing by possibly limiting access of sgRNAs to certain target sites. An additional challenge for genome editing is the abundance of multi-copy genes in the large *Symbiodinium* genomes. Gene redundancy can prevent knockout of gene function since the CRISPR/Cas9 system is not 100% efficient, meaning uncleaved functional gene copies can remain. Additionally, CRISPR/Cas9 targeting of genes with high copy numbers has been found to decrease cell proliferation and survival likely due to an increased frequency of DNA damage events (Aguirre et al., 2016). Also, design of sgRNAs requires a sequenced genome, but only three *Symbiodinium* genomes—each from a separate evolutionary lineage—are currently available.

As a first step to overcome some of these limitations, we analyzed the three published *Symbiodinium* genomes (types A1, B1, and F1; Shoguchi et al., 2013; Lin et al., 2015; Aranda et al., 2016) to identify conserved single copy genes. We then predicted a target site in each conserved gene with high sgRNA efficiency and specificity across the genomes (Supplementary Materials and Methods). Conserved target sites may permit CRISPR/Cas9 genome editing of *Symbiodinium* types that have yet to be sequenced. Our analysis revealed 1792 conserved single copy orthologs, 261 of which have an optimal target site compatible with all genomes (Supplementary Dataset 1a). The 261 single copy orthologs for CRISPR/Cas9 genome editing were enriched for a wide array of functional gene groups of interest, including cellular components for photosynthesis and biological pathways for oxidation-reduction and for response to UV-B (Supplementary Figure 1 and Supplementary Tables 2–4). Knockout of these genes would critically improve our understanding of *Symbiodinium* gene function, and if HDR is present in *Symbiodinium*, these sgRNA target sites could also be used to introduce genes of interest or entire *Symbiodinium* expression constructs into the genome. Furthermore, we identified sgRNA target sites in the type A1 genome scaffolds 710 and 484 (Aranda et al., 2016) immediately downstream from the potentially strong, constitutive *Symbiodinium* promoters discussed earlier (Supplementary Dataset 1b). Assuming HDR, reporter genes such as fluorescent proteins could be introduced at these sites to measure promoter activity.

The CRISPR/Cas9 system can be carried by plasmids that contain expression constructs for the Cas9, sgRNA, and in the case of HDR, the repair template with homologous arms. Target site cleavage is improved by increased CRISPR/Cas9 construct expression (Hsu et al., 2013), so strong endogenous promoters and terminators from *Symbiodinium* discussed earlier could be employed to drive transcription of Cas9 by *Symbiodinium*. However, transcription of sgRNAs requires RNA polymerase III (*Pol III*) rather than RNA polymerase II. Therefore, promoters specifically recognized by *Pol III* (e.g., promoter of the U6 snRNA gene) are needed. Such promoters have been isolated from other eukaryotes for sgRNA transcription; but, as discussed earlier, they contain motifs (e.g., TATA-box) that *Symbiodinium* lack (Goomer and Kunkel, 1992; Clarke et al., 2013). In *Symbiodinium*, 26 U6 snRNA gene copies have been identified (see Supplementary Table 5 in Shoguchi et al., 2013), one of which is unusually located in a cluster with U1, U2, U4, U5, 5S, and spliced leader snRNA genes (type B1 genome scaffold 8131; Shoguchi et al., 2013). Thus, genomic sequences found upstream and downstream of these *Symbiodinium* U6 snRNA genes could be isolated and trialed in sgRNA expression constructs as potential promoters and terminators recognized by *Symbiodinium Pol III*. Alternatively, the CRISPR/Cas9 system can be introduced to cells as pre-complexed sgRNA and purified Cas9 protein, which can achieve higher genome editing specificity by ∼10-fold compared to CRISPR/Cas9 plasmids and also removes the need to optimize Cas9 codon usage or to find appropriate promoters that will express Cas9 or sgRNAs (Zuris et al., 2015).

## Intracellular Delivery of Constructs and Complexes

Verified delivery of expression constructs into *Symbiodinium* was previously achieved using silicon carbide whiskers, which yielded very few transformants (ten Lohuis and Miller, 1998), and with *Agrobacterium*, which produced transient transformants that were unable to divide (Ortiz-Matamoros et al., 2015a). Low efficiency foreign DNA delivery may be due to obstruction by the thick, multilayer *Symbiodinium* cell covering comprised of an external polysaccharide or glycoprotein layer atop an internal cell wall (thecal plates and the pellicle) then finally the plasma membrane (Markell et al., 1992; Wakefield et al., 2000). To overcome this barrier, methods including high-voltage electroporation, bioballistics, microinjection, and viral transduction should be trialed. Continued exploration into *Symbiodinium* viruses may facilitate development of a compatible transduction system. Additionally, the first method to produce viable *Symbiodinium* protoplasts (cells with their cell wall removed) was developed (Levin et al., 2017b). Protoplasts have been instrumental in genetic manipulation of cell-walled organisms through somatic hybridization as well as by allowing for alternate DNA delivery methods (Davey et al., 2005). Protoplast-dependent methods such as polyethylene glycol-mediated transformation (Mathur and Koncz, 1998) and liposome-mediated transformation (Caboche, 1990) may improve efficiency of construct delivery into *Symbiodinium*. Cell walls also serve as a barrier to RNA/protein complexes like pre-complexed sgRNA and Cas9 protein. Thus, genome editing of *Symbiodinium* with pre-complexed sgRNA and Cas9 protein may require the use of protoplasts (Woo et al., 2015). Polyethylene glycol-mediated transformation (Woo et al., 2015), cationic lipid transformation (Zuris et al., 2015), and electroporation (Baek et al., 2016) have all been used to effectively deliver pre-complexed sgRNA and Cas9 protein through cell membranes of other eukaryotes that lacked cell walls.

## Can We Reduce Coral Bleaching with Genetically Enhanced *Symbiodinium?*

Coral reefs are the most diverse marine habitat per unit area (Reaka-Kudla et al., 1996; Knowlton et al., 2010) and provide world economies with nearly US$30 billion in net benefits from goods and services annually (Cesar et al., 2003). Climate change impact models predict that most reefs will be severely damaged or lost in this century unless immediate protection efforts are made (Hoegh-Guldberg et al., 2007; Pandolfi et al., 2011; Mora et al., 2016; Hughes et al., 2017) prompting calls for the development of novel mitigation and restoration approaches (Rinkevich, 2014; van Oppen et al., 2015, 2017; Piaggio et al., 2016). Exceptional genetic variability naturally exists within the genus *Symbiodinium*, suggesting that seeding vulnerable corals with more climate-change tolerant *Symbiodinium* variants could provide a means to reduce bleaching susceptibility of corals (van Oppen et al., 2015). Although, uptake of non-native *Symbiodinium* variants by corals may not be widely achievable since many coral species only associate with specific *Symbiodinium* types (LaJeunesse et al., 2004). Furthermore, shifts from innately less stress tolerant *Symbiodinium* types to more stress tolerant *Symbiodinium* types (e.g., from type C2 to D) can have negative impacts on a number of coral fitness traits including growth and fecundity (Little et al., 2004; Jones and Berkelmans, 2011).

Environmental bioengineering is an alternative strategy to safeguard against climate change (Solé, 2015; Piaggio et al., 2016). Microalgae, such as *Symbiodinium*, are clear and promising candidates for genetic engineering with the aim of regaining and preserving ecosystem-climate homeostasis (Solé, 2015) because they can significantly influence the health of entire ecosystems (Berkelmans and van Oppen, 2006; Kirk and Weis, 2016; Murray et al., 2016). Genetic engineering to increase stress tolerance of the *Symbiodinium* variants that are naturally harbored by at-risk corals holds potential to reduce bleaching susceptibility without negatively impacting the fitness of the coral host since existing *Symbiodinium*-coral partnerships would be preserved. *Fe-sod*, *Mn-sod Prxd*, and *Hsp70* genes from *Symbiodinium* (Levin et al., 2016; Gierz et al., 2017; Goyen et al., 2017) are standout candidates whose engineered up-regulation may enhance thermal and bleaching tolerance by reducing heat-induced oxidative damage, but thorough evaluation of how this artificial up-regulation contributes to long term fitness and the *Symbiodinium*-coral symbiosis would be mandatory.

Application of genetic engineering to support environmental management practices has been gaining momentum. Notably, sterile male mosquitoes have been engineered to control mosquito-borne diseases (Gabrieli et al., 2014). Field releases of the sterile males significantly reduced wild mosquito populations, supporting their value to disease control (Harris et al., 2012). Similarly, fungus-resistance has been engineered in American chestnut trees in order to restore the natural population that was nearly eradicated from the spread of a foreign fungus. Introduction of these transgenic trees into the wild may receive federal approval in just the next few years, which would make them the first threatened plant species to be restored through genetic engineering (Jacobs et al., 2013; Powell, 2014).

Considering the great promise shown by genetic engineering-based approaches to promote environmental health (Jacobs et al., 2013; Powell, 2014) and human health (Paine et al., 2005; Harris et al., 2012; Gabrieli et al., 2014), as well as to sustain food security (Schroeder et al., 2013), it is logical for genetic engineering to be proposed as an important component of the growing repertoire of forward-looking coral reef management approaches (van Oppen et al., 2015; Piaggio et al., 2016). Due to the urgent need to protect coral reefs from climate change, the *Symbiodinium* research community must commit to an all-hands-on-deck attitude to achieve and extensively test genetic enhancement of *Symbiodinium* and other novel reef restoration strategies in the laboratory setting. In parallel, comprehensive cost-benefit-risk evaluation of the potential ecological and socioeconomic impacts from implementation of such strategies in the natural environment must be exhaustive before field-based trials are initiated. Additionally, transparent dialogs with policy makers, coral reef managers, and the general public need to be initiated now to begin the process of education and public acceptance of genetic engineering approaches for coral reef mitigation and restoration.

As we have discussed here, recent NGS breakthroughs have revealed natural genetic elements of *Symbiodinium* and their viruses (**Figure 1**). Based on these discoveries, we have developed a tailored genetic engineering framework for *Symbiodinium* based on empirical data that may also be applicable to other dinoflagellate genera. In doing so, we have opened a new prospective avenue to decode *Symbiodinium* functional genomics that may ultimately allow for engineering increased stress tolerance of *Symbiodinium* to reduce coral bleaching.

## Author Contributions

RL conceived the manuscript concept, analyzed NGS data, and wrote the manuscript. CRV analyzed NGS data and critically edited the theory and writing of the manuscript. SA analyzed NGS data. PS, DS, and MvO critically edited the theory and writing of the manuscript.

## Conflict of Interest Statement

The authors declare that the research was conducted in the absence of any commercial or financial relationships that could be construed as a potential conflict of interest. The reviewer AWL declared a shared affiliation, though no other collaboration, with one of the authors DS to the handling Editor, who ensured that the process nevertheless met the standards of a fair and objective review.

## Footnotes

1 http://www.symbiogbr.org/

2 https://sustainabledevelopment.un.org/?menu=1300, last accessed March 2017.

## References

[B1] Aguirre A. J. Meyers R. M. Weir B. A. Vazquez F. Zhang C.-Z. Ben-David U. (2016). Genomic copy number dictates a gene-independent cell response to CRISPR-Cas9 targeting. *Cancer Discov.* 6 914–929. 10.1158/2159-8290.CD-16-0154 27260156 PMC4972686

[B2] Alavi M. Miller T. Erlandson K. Schneider R. Belas R. (2001). Bacterial community associated with Pfiesteria-like dinoflagellate cultures. *Environ. Microbiol.* 3 380–396. 10.1046/j.1462-2920.2001.00207.x 11472503

[B3] Allera-Moreau C. Delluc-Clavières A. Castano C. Van den Berghe L. Golzio M. Moreau M. (2007). Long term expression of bicistronic vector driven by the FGF-1 IRES in mouse muscle. *BMC Biotechnol.* 7:74 10.1186/1472-6750-7-74 PMC2180170 17963525

[B4] Aranda M. Li Y. Liew Y. Baumgarten S. Simakov O. Wilson M. (2016). Genomes of coral dinoflagellate symbionts highlight evolutionary adaptations conducive to a symbiotic lifestyle. *Sci. Rep.* 6:39734 10.1038/srep39734 PMC5177918 28004835

[B5] Arif C. Daniels C. Bayer T. Banguera-Hinestroza E. Barbrook A. Howe C. J. (2014). Assessing *Symbiodinium* diversity in scleractinian corals via next-generation sequencing-based genotyping of the ITS2 rDNA region. *Mol. Ecol.* 23 4418–4433. 10.1111/mec.12869 25052021 PMC4285332

[B6] Bachvaroff T. R. Place A. R. (2008). From stop to start: tandem gene arrangement, copy number and trans-splicing sites in the dinoflagellate *Amphidinium carterae*. *PLoS ONE* 3:e2929 10.1371/journal.pone.0002929 PMC2488372 18698341

[B7] Baek K. Kim D. H. Jeong J. Sim S. J. Melis A. Kim J.-S. (2016). DNA-free two-gene knockout in *Chlamydomonas reinhardtii* via CRISPR-Cas9 ribonucleoproteins. *Sci. Rep.* 6:30620 10.1038/srep30620 PMC4964356 27466170

[B8] Barshis D. J. Ladner J. T. Oliver T. A. Palumbi S. R. (2014). Lineage-specific transcriptional profiles of *Symbiodinium* spp. unaltered by heat stress in a coral host. *Mol. Biol. Evol.* 31 1343–1352. 10.1093/molbev/msu107 24651035

[B9] Baumgarten S. Bayer T. Aranda M. Liew Y. J. Carr A. Micklem G. (2013). Integrating microRNA and mRNA expression profiling in *Symbiodinium microadriaticum*, a dinoflagellate symbiont of reef-building corals. *BMC Genomics* 14:704 10.1186/1471-2164-14-704 PMC3853145 24119094

[B10] Bayer T. Aranda M. Sunagawa S. Yum L. K. DeSalvo M. K. Lindquist E. (2012). *Symbiodinium* transcriptomes: genome insights into the dinoflagellate symbionts of reef-building corals. *PLoS ONE* 7:e35269 10.1371/journal.pone.0035269 PMC3329448 22529998

[B11] Benfey P. N. Chua N.-H. (1990). The cauliflower mosaic virus 35S promoter: combinatorial regulation of transcription in plants. *Science* 250 959–966. 10.1126/science.250.4983.959 17746920

[B12] Berkelmans R. van Oppen M. J. (2006). The role of zooxanthellae in the thermal tolerance of corals: a ‘nugget of hope’for coral reefs in an era of climate change. *Proc. R. Soc. Lond. B Biol. Sci.* 273 2305–2312. 10.1098/rspb.2006.3567 PMC1636081 16928632

[B13] Boulotte N. M. Dalton S. J. Carroll A. G. Harrison P. L. Putnam H. M. Peplow L. M. (2016). Exploring the *Symbiodinium* rare biosphere provides evidence for symbiont switching in reef-building corals. *ISME J.* 10 2693–2701. 10.1038/ismej.2016.54 27093048 PMC5113844

[B14] Breyer D. Kopertekh L. Reheul D. (2014). Alternatives to antibiotic resistance marker genes for in vitro selection of genetically modified plants–scientific developments, current use, operational access and biosafety considerations. *Crit. Rev. Plant Sci.* 33 286–330. 10.1080/07352689.2013.870422

[B15] Byler K. A. Carmi-Veal M. Fine M. Goulet T. L. (2013). Multiple symbiont acquisition strategies as an adaptive mechanism in the coral *Stylophora pistillata*. *PLoS ONE* 8:e59596 10.1371/journal.pone.0059596 PMC3608662 23555721

[B16] Caboche M. (1990). Liposome-mediated transfer of nucleic acids in plant protoplasts. *Physiol. Plant.* 79 173–176. 10.1111/j.1399-3054.1990.tb05882.x

[B17] Cesar H. Burke L. Pet-Soede L. (2003). *The Economics of Worldwide Coral Reef Degradation.* Arnhem: Cesar Environmental Economics Consulting (CEEC).

[B18] Chi J. Parrow M. W. Dunthorn M. (2014). Cryptic sex in *Symbiodinium* (Alveolata, Dinoflagellata) is supported by an inventory of meiotic genes. *J. Eukaryot. Microbiol.* 61 322–327. 10.1111/jeu.12110 24904932

[B19] Chu V. T. Weber T. Wefers B. Wurst W. Sander S. Rajewsky K. (2015). Increasing the efficiency of homology-directed repair for CRISPR-Cas9-induced precise gene editing in mammalian cells. *Nat. Biotechnol.* 33 543–548. 10.1038/nbt.3198 25803306

[B20] Clarke B. D. Cummins D. M. McColl K. A. Ward A. C. Doran T. J. (2013). Characterization of zebrafish polymerase III promoters for the expression of short-hairpin RNA interference molecules. *Zebrafish* 10 472–479. 10.1089/zeb.2012.0782 23030845

[B21] Coffroth M. A. Santos S. R. (2005). Genetic diversity of symbiotic dinoflagellates in the genus *Symbiodinium*. *Protist* 156 19–34. 10.1016/j.protis.2005.02.004 16048130

[B22] Colanduoni J. A. Villafranca J. J. (1986). Inhibition of *Escherichia coli* glutamine synthetase by phosphinothricin. *Bioorg. Chem.* 14 163–169. 10.1016/0045-2068(86)90026-X

[B23] Cong L. Ran F. A. Cox D. Lin S. Barretto R. Habib N. (2013). Multiplex genome engineering using CRISPR/Cas systems. *Science* 339 819–823. 10.1126/science.1231143 23287718 PMC3795411

[B24] Correa A. M. Ainsworth T. D. Rosales S. M. Thurber A. R. Butler C. R. Thurber R. L. V. (2016). Viral outbreak in corals associated with an in situ bleaching event: atypical herpes-like viruses and a new megavirus infecting *Symbiodinium*. *Front. Microbiol.* 7:127 10.3389/fmicb.2016.00127 PMC4761846 26941712

[B25] Correa A. M. Welsh R. M. Thurber R. L. V. (2013). Unique nucleocytoplasmic dsDNA and &plus; ssRNA viruses are associated with the dinoflagellate endosymbionts of corals. *ISME J.* 7 13–27. 10.1038/ismej.2012.75 22791238 PMC3526182

[B26] Croft M. T. Lawrence A. D. Raux-Deery E. Warren M. J. Smith A. G. (2005). Algae acquire vitamin B12 through a symbiotic relationship with bacteria. *Nature* 438 90–93. 10.1038/nature04056 16267554

[B27] Davey M. R. Anthony P. Power J. B. Lowe K. C. (2005). Plant protoplast technology: current status. *Acta Physiol. Plant.* 27 117–130. 10.1007/s11738-005-0044-0

[B28] DeSalvo M. K. Sunagawa S. Fisher P. L. Voolstra C. R. Iglesias-Prieto R. Medina M. (2010). Coral host transcriptomic states are correlated with *Symbiodinium* genotypes. *Mol. Ecol* 19 1174–1186. 10.1111/j.1365-294X.2010.04534.x 20149089

[B29] Edmunds P. J. Pochon X. Levitan D. R. Yost D. M. Belcaid M. Putnam H. M. (2014). Long-term changes in *Symbiodinium* communities in *Orbicella annularis* in St. John, US Virgin Islands. *Mar. Ecol. Prog. Ser.* 506 129–144. 10.3354/meps10808

[B30] Figueroa R. I. Dapena C. Bravo I. Cuadrado A. (2015). The hidden sexuality of alexandrium minutum: an example of overlooked sex in Dinoflagellates. *PLoS ONE* 10:e0142667 10.1371/journal.pone.0142667 PMC4979955 26599692

[B31] Gabrieli P. Smidler A. Catteruccia F. (2014). Engineering the control of mosquito-borne infectious diseases. *Genome Biol.* 15 535 10.1186/s13059-014-0535-7 PMC4282146 25418061

[B32] Gierz S. L. Forêt S. Leggat W. (2017). Transcriptomic analysis of thermally stressed *Symbiodinium* reveals differential expression of stress and metabolism genes. *Front. Plant Sci.* 8:271 10.3389/fpls.2017.00271 PMC5328969 28293249

[B33] Gonzalez A. Jimenez A. Vazquez D. Davies J. Schindler D. (1978). Studies on the mode of action of hygromycin B, an inhibitor of translocation in eukaryotes. *Biochim. Biophys. Acta* 521 459–469. 10.1016/0005-2787(78)90287-3 367435

[B34] Goomer R. Kunkel G. (1992). The transcriptional start site for a human U6 small nuclear RNA gene is dictated by a compound promoter element consisting of the PSE and the TATA box. *Nucleic Acids Res.* 20 4903–4912. 10.1093/nar/20.18.4903 1408805 PMC334249

[B35] Goyen S. Pernice M. Szabó M. Warner M. E. Ralph P. J. Suggett D. J. (2017). A molecular physiology basis for functional diversity of hydrogen peroxide production amongst *Symbiodinium* spp.(*Dinophyceae)*. *Mar. Biol.* 164 46 10.1007/s00227-017-3073-5

[B36] Gurtu V. Yan G. Zhang G. (1996). IRES bicistronic expression vectors for efficient creation of stable mammalian cell lines. *Biochem. Biophys. Res. Commun.* 229 295–298. 10.1006/bbrc.1996.1795 8954121

[B37] Harris A. F. McKemey A. R. Nimmo D. Curtis Z. Black I. Morgan S. A. (2012). Successful suppression of a field mosquito population by sustained release of engineered male mosquitoes. *Nat. Biotechnol.* 30 828–830. 10.1038/nbt.2350 22965050

[B38] Hennige S. Suggett D. J. Warner M. E. McDougall K. Smith D. J. (2009). Photobiology of *Symbiodinium* revisited: bio-physical and bio-optical signatures. *Coral Reefs* 28 179–195. 10.1007/s00338-008-0444-x

[B39] Hoegh-Guldberg O. Mumby P. J. Hooten A. J. Steneck R. S. Greenfield P. Gomez E. (2007). Coral reefs under rapid climate change and ocean acidification. *Science* 318 1737–1742. 10.1126/science.1152509 18079392

[B40] Holloman W. K. (2011). Unraveling the mechanism of BRCA2 in homologous recombination. *Nat. Struct. Mol. Biol.* 18 748–754. 10.1038/nsmb.2096 21731065 PMC3647347

[B41] Hsu P. D. Scott D. A. Weinstein J. A. Ran F. A. Konermann S. Agarwala V. (2013). DNA targeting specificity of RNA-guided Cas9 nucleases. *Nat. Biotechnol.* 31 827–832. 10.1038/nbt.2647 23873081 PMC3969858

[B42] Hughes T. P. Kerry J. T. Álvarez-Noriega M. Álvarez-Romero J. G. Anderson K. D. Baird A. H. (2017). Global warming and recurrent mass bleaching of corals. *Nature* 543 373–377. 10.1038/nature21707 28300113

[B43] Jacobs D. F. Dalgleish H. J. Nelson C. D. (2013). A conceptual framework for restoration of threatened plants: the effective model of American chestnut (*Castanea dentata*) reintroduction. *New Phytol.* 197 378–393. 10.1111/nph.12020 23163342

[B44] Jiang J. Zhang H. Kang Y. Bina D. Lo C. S. Blankenship R. E. (2012). Characterization of the peridinin–chlorophyll a-protein complex in the dinoflagellate *Symbiodinium*. *Biochim. Biophys. Acta* 1817 983–989. 10.1016/j.bbabio.2012.03.027 22497797 PMC3947849

[B45] Jones A. M. Berkelmans R. (2011). Tradeoffs to thermal acclimation: energetics and reproduction of a reef coral with heat tolerant *Symbiodinium* type-D. *J. Mar. Biol.* 2011 12 10.1371/journal.pone.0010437

[B46] Karas B. J. Diner R. E. Lefebvre S. C. McQuaid J. Phillips A. P. Noddings C. M. (2015). Designer diatom episomes delivered by bacterial conjugation. *Nat. Commun.* 6 1–10. 10.1038/ncomms7925 PMC4411287 25897682

[B47] Kirk N. L. Weis V. M. (2016). “Animal–*Symbiodinium* symbioses: foundations of coral reef ecosystems,” in *The Mechanistic Benefits of Microbial Symbionts*, ed. Hurst C. J. (Cham: Springer), 269–294. 10.1007/978-3-319-28068-4_10

[B48] Knowlton N. Brainard R. E. Fisher R. Moews M. Plaisance L. Caley M. J. (2010). “Coral reef biodiversity,” in *Life in the World’s Oceans: Diversity Distribution and Abundance*, ed. Mcintyre A. D. (Hoboken, NJ: John Wiley & Sons), 65–74. 10.1002/9781444325508.ch4

[B49] Krueger T. Fisher P. L. Becker S. Pontasch S. Dove S. Hoegh-Guldberg O. (2015). Transcriptomic characterization of the enzymatic antioxidants FeSOD, MnSOD, APX and KatG in the dinoflagellate genus *Symbiodinium*. *BMC Evol. Biol.* 15:48 10.1186/s12862-015-0326-0 PMC4416395 25887897

[B50] Ladner J. T. Barshis D. J. Palumbi S. R. (2012). Protein evolution in two co-occurring types of *Symbiodinium*: an exploration into the genetic basis of thermal tolerance in *Symbiodinium* clade D. *BMC Evol. Biol.* 12:217 10.1186/1471-2148-12-217 PMC3740780 23145489

[B51] LaJeunesse T. C. Thornhill D. J. Cox E. F. Stanton F. G. Fitt W. K. Schmidt G. W. (2004). High diversity and host specificity observed among symbiotic dinoflagellates in reef coral communities from Hawaii. *Coral Reefs* 23 596–603. 10.1007/s00338-004-0428-4

[B52] Lee S. E. Pâques F. Sylvan J. Haber J. E. (1999). Role of yeast SIR genes and mating type in directing DNA double-strand breaks to homologous and non-homologous repair paths. *Curr. Biol.* 9 767–770. 10.1016/S0960-9822(99)80339-X 10421582

[B53] Leggat W. Yellowlees D. Medina M. (2011). Recent progress in *Symbiodinium* transcriptomics. *J. Exp. Mar. Biol. Ecol.* 408 120–125. 10.1016/j.jembe.2011.07.032

[B54] Levin R. A. Beltran V. H. Hill R. Kjelleberg S. McDougald D. Steinberg P. D. (2016). Sex, scavengers, and chaperones: transcriptome secrets of divergent symbiodinium thermal tolerances. *Mol. Biol. Evol.* 33 2201–2215. 10.1093/molbev/msw119 27301593 PMC4989115

[B55] Levin R. A. Felsen C. N. Yang J. Lin J. Y. Whitney M. A. Nguyen Q. T. (2014). An optimized triple modality reporter for quantitative in vivo tumor imaging and therapy evaluation. *PLoS ONE* 9:e97415 10.1371/journal.pone.0097415 PMC4016317 24816650

[B56] Levin R. A. Suggett D. J. Nitschke M. R. van Oppen M. J. Steinberg P. D. (2017b). Expanding the *Symbiodinium* (Dinophyceae, Suessiales) toolkit through protoplast technology. *J. Eukaryot. Microbiol.* 10.1111/jeu.12393 [Epub ahead of print]. 28120360

[B57] Levin R. A. Voolstra C. R. Weynberg K. D. van Oppen M. J. H. (2017a). Evidence for a role of viruses in the thermal sensitivity of coral photosymbionts. *ISME J.* 11 808–812. 10.1038/ismej.2016.154 27911439 PMC5322306

[B58] Lin S. Cheng S. Song B. Zhong X. Lin X. Li W. (2015). The *Symbiodinium* kawagutii genome illuminates dinoflagellate gene expression and coral symbiosis. *Science* 350 691–694. 10.1126/science.aad0408 26542574

[B59] Little A. F. Van Oppen M. J. Willis B. L. (2004). Flexibility in algal endosymbioses shapes growth in reef corals. *Science* 304 1492–1494. 10.1126/science.1095733 15178799

[B60] Markell D. Trench R. Iglesias-Prieto R. (1992). Macromolecules associated with the cell walls of symbiotic dinoflagellates. *Symbiosis* 12 19–31.

[B61] Martínez-Salas E. (1999). Internal ribosome entry site biology and its use in expression vectors. *Curr. Opin. Biotechnol.* 10 458–464. 10.1016/S0958-1669(99)00010-5 10508627

[B62] Mathur J. Koncz C. (1998). PEG-mediated protoplast transformation with naked DNA. *Methods Mol. Biol.* 82 267–276. 10.1385/0-89603-391-0:267 9664432

[B63] Miller T. R. Belas R. (2006). Motility is involved in Silicibacter sp. TM1040 interaction with dinoflagellates. *Environ. Microbiol.* 8 1648–1659. 10.1111/j.1462-2920.2006.01071.x 16913924

[B64] Moldowan J. M. Talyzina N. M. (1998). Biogeochemical evidence for dinoflagellate ancestors in the Early Cambrian. *Science* 281 1168–1170. 10.1126/science.281.5380.1168 9712575

[B65] Mora C. Graham N. A. Nyström M. (2016). Ecological limitations to the resilience of coral reefs. *Coral Reefs* 35 1271–1280. 10.1007/s00338-016-1479-z

[B66] Murray S. A. Suggett D. J. Doblin M. A. Kohli G. S. Seymour J. R. Fabris M. (2016). Unravelling the functional genetics of dinoflagellates: a review of approaches and opportunities. *Perspect. Phycol.* 3 37–52. 10.1127/pip/2016/0039

[B67] Muscatine L. (1990). The role of symbiotic algae in carbon and energy flux in reef corals. *Ecosyst. World* 25 75–87.

[B68] Muscatine L. Porter J. W. (1977). Reef corals: mutualistic symbioses adapted to nutrient-poor environments. *Bioscience* 27 454–460. 10.2307/1297526

[B69] Nehlsen K. Broll S. Bode J. (2006). Replicating minicircles: generation of nonviral episomes for the efficient modification of dividing cells. *Gene Ther. Mol. Biol.* 10 233–244.

[B70] Nymark M. Sharma A. K. Sparstad T. Bones A. M. Winge P. (2016). A CRISPR/Cas9 system adapted for gene editing in marine algae. *Sci. Rep.* 6:24951 10.1038/srep24951 PMC4842962 27108533

[B71] Ortiz-Matamoros M. F. Islas-Flores T. Voigt B. Menzel D. Baluška F. Villanueva M. A. (2015a). Heterologous DNA uptake in cultured *Symbiodinium* spp. Aided by *Agrobacterium tumefaciens*. *PLoS ONE* 10:e0132693 10.1371/journal.pone.0132693 PMC4500500 26167858

[B72] Ortiz-Matamoros M. F. Villanueva M. A. Islas-Flores T. (2015b). Transient transformation of cultured photosynthetic dinoflagellates (*Symbiodinium* spp.) with plant-targeted vectors Transformación de dinoflagelados fotosintéticos del género *Symbiodinium* en cultivo con vectores diseñados para plantas. *Ciencias Marinas* 41 21–32. 10.7773/cm.v41i1.2449

[B73] Paine J. A. Shipton C. A. Chaggar S. Howells R. M. Kennedy M. J. Vernon G. (2005). Improving the nutritional value of Golden Rice through increased pro-vitamin A content. *Nat. Biotechnol.* 23 482–487. 10.1038/nbt1082 15793573

[B74] Pandolfi J. M. Connolly S. R. Marshall D. J. Cohen A. L. (2011). Projecting coral reef futures under global warming and ocean acidification. *Science* 333 418–422. 10.1126/science.1204794 21778392

[B75] Parkinson J. E. Baumgarten S. Michell C. T. Baums I. B. LaJeunesse T. C. Voolstra C. R. (2016). Gene expression variation resolves species and individual strains among coral-associated dinoflagellates within the genus *Symbiodinium*. *Genome Biol. Evol.* 8 665–680. 10.1093/gbe/evw019 26868597 PMC4824173

[B76] Piaggio A. J. Segelbacher G. Seddon P. J. Alphey L. Bennett E. L. Carlson R. H. (2016). Is it time for synthetic biodiversity conservation? *Trends Ecol. Evol.* 32 97–107. 10.1016/j.tree.2016.10.016 27871673

[B77] Pline W. A. Lacy G. H. Stromberg V. Hatzios K. K. (2001). Antibacterial activity of the herbicide glufosinate on *Pseudomonas syringae* pathovar glycinea. *Pestic. Biochem. Physiol.* 71 48–55. 10.1006/pest.2001.2556

[B78] Pochon X. Gates R. D. (2010). A new *Symbiodinium* clade (Dinophyceae) from soritid foraminifera in Hawai’i. *Mol. Phylogenet. Evol.* 56 492–497. 10.1016/j.ympev.2010.03.040 20371383

[B79] Pochon X. Montoya-Burgos J. I. Stadelmann B. Pawlowski J. (2006). Molecular phylogeny, evolutionary rates, and divergence timing of the symbiotic dinoflagellate genus *Symbiodinium*. *Mol. Phylogenet. Evol.* 38 20–30. 10.1016/j.ympev.2005.04.028 15978847

[B80] Powell W. (2014). The american chestnut’s genetic rebirth. *Sci. Am.* 310 68–73. 10.1038/scientificamerican0314-68 24660331

[B81] Quigley K. M. Davies S. W. Kenkel C. D. Willis B. L. Matz M. V. Bay L. K. (2014). Deep-sequencing method for quantifying background abundances of *Symbiodinium* types: exploring the rare *Symbiodinium* biosphere in reef-building corals. *PLoS ONE* 9:e94297 10.1371/journal.pone.0094297 PMC3984134 24728373

[B82] Ran F. A. Hsu P. D. Wright J. Agarwala V. Scott D. A. Zhang F. (2013). Genome engineering using the CRISPR-Cas9 system. *Nat. Protoc.* 8 2281–2308. 10.1038/nprot.2013.143 24157548 PMC3969860

[B83] Reaka-Kudla M. L. Wilson D. E. Wilson E. O. (1996). *Biodiversity II: Understanding and Protecting our Biological Resources.* Washington, DC: Joseph Henry Press.

[B84] Rinkevich B. (2014). Rebuilding coral reefs: does active reef restoration lead to sustainable reefs? *Curr. Opin. Environ. Sustain.* 7 28–36. 10.1016/j.cosust.2013.11.018

[B85] Ritchie K. B. (2012). “Bacterial symbionts of corals and *Symbiodinium*,” in *Beneficial Microorganisms in Multicellular Life Forms*, eds Rosenberg E. Gophna U. (Berlin: Springer), 139–150. 10.1007/978-3-642-21680-0_9

[B86] Rosic N. Ling E. Y. S. Chan C.-K. K. Lee H. C. Kaniewska P. Edwards D. (2015). Unfolding the secrets of coral–algal symbiosis. *ISME J.* 9 844–856. 10.1038/ismej.2014.182 25343511 PMC4817714

[B87] Santos S. R. Coffroth M. A. (2003). Molecular genetic evidence that dinoflagellates belonging to the genus *Symbiodinium* Freudenthal are haploid. *Biol. Bull.* 204 10–20. 10.2307/1543491 12588740

[B88] Schroeder J. I. Delhaize E. Frommer W. B. Guerinot M. L. Harrison M. J. Herrera-Estrella L. (2013). Using membrane transporters to improve crops for sustainable food production. *Nature* 497 60–66. 10.1038/nature11909 23636397 PMC3954111

[B89] Shaner N. C. Steinbach P. A. Tsien R. Y. (2005). A guide to choosing fluorescent proteins. *Nat. Methods* 2 905–909. 10.1038/nmeth819 16299475

[B90] Shoguchi E. Shinzato C. Hisata K. Satoh N. Mungpakdee S. (2015). The large mitochondrial genome of *Symbiodinium* minutum reveals conserved noncoding sequences between Dinoflagellates and Apicomplexans. *Genome Biol. Evol.* 7 2237–2244. 10.1093/gbe/evv137 26199191 PMC4558855

[B91] Shoguchi E. Shinzato C. Kawashima T. Gyoja F. Mungpakdee S. Koyanagi R. (2013). Draft assembly of the *Symbiodinium* minutum nuclear genome reveals dinoflagellate gene structure. *Curr. Biol.* 23 1399–1408. 10.1016/j.cub.2013.05.062 23850284

[B92] Solé R. (2015). Bioengineering the biosphere? *Ecol. Complex.* 22 40–49. 10.1016/j.ecocom.2015.01.005

[B93] Stoykova P. Stoeva-Popova P. (2011). PMI (manA) as a nonantibiotic selectable marker gene in plant biotechnology. *Plant Cell Tissue Organ Cult. (PCTOC)* 105 141–148. 10.1007/s11240-010-9858-6

[B94] Suggett D. J. Warner M. E. Smith D. J. Davey P. Hennige S. Baker N. R. (2008). Photosynthesis and production of hydrogen peroxide by *symbiodinium* (Pyrrophyta) phylotypes with different thermal tolerances. *J Phycol.* 44 948–956. 10.1111/j.1529-8817.2008.00537.x 27041613

[B95] ten Lohuis M. R. Miller D. J. (1998). Genetic transformation of dinoflagellates (Amphidinium and *Symbiodinium*): expression of GUS in microalgae using heterologous promoter constructs. *Plant J.* 13 427–435. 10.1046/j.1365-313X.1998.00040.x

[B96] Thacker D. Keeney S. (2016). “Homologous recombination during meiosis,” in *DNA Replication, Recombination, and Repair*, eds Hanaoka F. Sugasawa K. (Tokyo: Springer), 131–151. 10.1007/978-4-431-55873-6_6

[B97] Thomas L. Kendrick G. Kennington W. Richards Z. Stat M. (2014). Exploring *Symbiodinium* diversity and host specificity in Acropora corals from geographical extremes of Western Australia with 454 amplicon pyrosequencing. *Mol. Ecol.* 23 3113–3126. 10.1111/mec.12801 24845644

[B98] Toller W. W. Rowan R. Knowlton N. (2001). Repopulation of zooxanthellae in the Caribbean corals *Montastraea annularis* and *M. faveolata* following experimental and disease-associated bleaching. *Biol. Bull.* 201 360–373. 10.2307/1543614 11751248

[B99] Tonk L. Bongaerts P. Sampayo E. M. Hoegh-Guldberg O. (2013). SymbioGBR: a web-based database of *Symbiodinium* associated with cnidarian hosts on the Great Barrier Reef. *BMC Ecol.* 13:7 10.1186/1472-6785-13-7 PMC3616961 23497177

[B100] Trench R. K. Blank R. J. (1987). *Symbiodinium* Microadriaticum Freudenthal, *S. Goreauii* Sp. Nov., *S. Kawagutii* Sp. Nov. and *S. Pilosum* Sp. Nov.: Gymnodinioid Dinoflagellate symbionts of marine invertebrates 1. *J. Phycol.* 23 469–481. 10.1111/j.1529-8817.1987.tb02534.x

[B101] van Oppen M. J. Gates R. D. Blackall L. L. Cantin N. Chakravarti L. J. Chan W. Y. (2017). Shifting paradigms in restoration of the world’s coral reefs. *Glob. Chang Biol.* 10.1111/gcb.13647 [Epub ahead of print]. 28247459

[B102] van Oppen M. J. H. Oliver J. K. Putnam H. M. Gates R. D. (2015). Building coral reef resilience through assisted evolution. *Proc. Natl. Acad. Sci. U.S.A.* 112 2307–2313. 10.1073/pnas.1422301112 25646461 PMC4345611

[B103] van Oppen M. J. H. Palstra F. P. Piquet A. M. T. Miller D. J. (2001). Patterns of coral–dinoflagellate associations in Acropora: significance of local availability and physiology of *Symbiodinium* strains and host–symbiont selectivity. *Proc. R. Soc. Lond. B. Biol. Sci.* 268 1759–1767. 10.1098/rspb.2001.1733 PMC1088806 11522193

[B104] Vicens Q. Westhof E. (2003). Crystal structure of geneticin bound to a bacterial 16S ribosomal RNA A site oligonucleotide. *J. Mol. Biol.* 326 1175–1188. 10.1016/S0022-2836(02)01435-3 12589761

[B105] Wakefield T. S. Farmer M. A. Kempf S. C. (2000). Revised description of the fine structure of in situ” zooxanthellae” genus *Symbiodinium*. *Biol. Bull.* 199 76–84. 10.2307/1542709 10975645

[B106] Warner M. E. Fitt W. K. Schmidt G. W. (1999). Damage to photosystem II in symbiotic dinoflagellates: a determinant of coral bleaching. *Proc. Natl. Acad. Sci. U.S.A.* 96 8007–8012. 10.1073/pnas.96.14.8007 10393938 PMC22178

[B107] Watrin E. Legagneux V. (2003). Introduction to chromosome dynamics in mitosis. *Biol. Cell* 95 507–513. 10.1016/j.biolcel.2003.08.003 14630387

[B108] Weis V. M. (2008). Cellular mechanisms of Cnidarian bleaching: stress causes the collapse of symbiosis. *J. Exp. Biol.* 211 3059–3066. 10.1242/jeb.009597 18805804

[B109] Weynberg K. D. Neave M. Clode P. L. Voolstra C. R. Brownlee C. Laffy P. (2017). Prevalent and persistent viral infection in cultures of the coral algal endosymbiont *Symbiodinium*. *Coral Reefs* 2017 1–12. 10.1007/s00338-017-1568-7

[B110] Weynberg K. D. Wood-Charlson E. M. Suttle C. A. van Oppen M. J. (2014). Generating viral metagenomes from the coral holobiont. *Front. Microbiol.* 5:206 10.3389/fmicb.2014.00206 PMC4019844 24847321

[B111] Wilkinson S. P. Fisher P. L. van Oppen M. J. H. Davy S. K. (2015). Intra-genomic variation in symbiotic dinoflagellates: recent divergence or recombination between lineages? *BMC Evol. Biol.* 15:46 10.1186/s12862-015-0325-1 PMC4381663 25887753

[B112] Woo J. W. Kim J. Kwon S. I. Corvalán C. Cho S. W. Kim H. (2015). DNA-free genome editing in plants with preassembled CRISPR-Cas9 ribonucleoproteins. *Nat. Biotechnol.* 33 1162–1164. 10.1038/nbt.3389 26479191

[B113] Xiang T. Nelson W. Rodriguez J. Tolleter D. Grossman A. R. (2015). *Symbiodinium* transcriptome and global responses of cells to immediate changes in light intensity when grown under autotrophic or mixotrophic conditions. *Plant J.* 82 67–80. 10.1111/tpj.12789 25664570

[B114] Yuyama I. Harii S. Hidaka M. (2012). Algal symbiont type affects gene expression in juveniles of the coral Acropora tenuis exposed to thermal stress. *Mar. Environ. Res.* 76 41–47. 10.1016/j.marenvres.2011.09.004 22001189

[B115] Zhang H. Zhuang Y. Gill J. Lin S. (2013). Proof that dinoflagellate spliced leader (DinoSL) is a useful hook for fishing dinoflagellate transcripts from mixed microbial samples: *Symbiodinium* kawagutii as a case study. *Protist* 164 510–527. 10.1016/j.protis.2013.04.002 23773861

[B116] Zuris J. A. Thompson D. B. Shu Y. Guilinger J. P. Bessen J. L. Hu J. H. (2015). Cationic lipid-mediated delivery of proteins enables efficient protein-based genome editing in vitro and in vivo. *Nat. Biotechnol.* 33 73–80. 10.1038/nbt.3081 25357182 PMC4289409

