# Supplementary material for: Engineering Strategies to Decode and Enhance the Genomes of Coral Symbionts
Source: Front Microbiol. 2017 Jun 30;8:1220. doi: 10.3389/fmicb.2017.01220 (PMC5492045; doi:10.3389/fmicb.2017.01220)
Supplement: Supplementary file 1 [file Supplementary_Information.PDF]

SUPPLEMENTARY MATERIALS AND METHODS

sgRNA design for functional genomics studies

Orthologous genes (n = 1,792) between three *Symbiodinium* types (A1, B1, and F1) were identified using the software Proteinortho v5.13 (Lechner et al., 2011) using genes predicted from their respective genomes (Shoguchi et al., 2013; Lin et al., 2015; Aranda et al., 2016). Genes found in type A1 (*S. microadriaticum*) that had orthologs in all three types and no paralogs in the intra- or inter-specific comparisons (i.e., 1-to-1 orthologs) were selected for designing sgRNAs using the R package CRISPRseek (Zhu et al., 2014). The sgRNA design and quality control for specificity and efficiency was determined as follows: CRISPRseek searched each ortholog for an optimal target sgRNA sequence approximately ~20 bp in length, the sequences of these potential sgRNA candidates were then compared to the entire type A1 genome to estimate specificity and efficiency of the sgRNA towards the target gene. The sgRNAs were ranked and ordered on the basis of their overall score and sgRNA efficiency. Out of the 1,792 genes, sgRNAs could be designed for 1,789 genes.

To ensure that sgRNAs for a particular gene in type A1 were specific to their corresponding orthologs in the other two species, BLASTn searches were carried out with all potential sgRNAs (n = 351,691, orthologs = 1,789) queried against an ortholog database from type B1 (*S. minutum*) and type F1 (*S. kawagutii*). Out of the 351,691 sgRNAs designed for 1,789 orthologs from type A1, 11,480 sgRNAs found hits to 1,208 orthologs in type B1. Similarly, 11,969 sgRNAs found hits for 1,115 orthologs in type F1. Given that our primary aim was to identify sgRNAs that can be used to target the same ortholog in all three species, 2,805 sgRNAs from 574 genes that had matches in all three types were used for further analyses. Besides type A1, these sgRNAs were scored for target specificity and efficiency against the genomes of type B1 and type F1 in separate runs of CRISPRseek with a maximum of four mismatches allowed between sgRNAs and the target sequence. Out of the initial 2,805 sgRNAs covering 574 orthologs, 2,757 (572 orthologs) could be successfully scored for type B1 and 2,782 sgRNAs (574 orthologs) could be successfully scored for type F1.

The BLASTn filtering above allowed filtering for sgRNA sequences that match the intended target gene, but did not preclude cases where short sequence stretches of sgRNAs had matches to non-target genes. To gauge the efficiency of sgRNA to the intended target gene, all flanking sequences identified for a particular sgRNA and species were run through BLAST (e-value < 10e-5, percent identity = 100%) and only those with a correct sgRNA-ortholog match were kept. This procedure resulted in 2,244 sgRNAs targeting 495 orthologs in type A1, 1,335 sgRNAs targeting 376 genes in type B1, and 1,582 sgRNAs targeting 419 orthologs in type F1. Among these, 825 sgRNAs targeting 261 orthologs were common to all three species and one sgRNA per ortholog

(261 sgRNA for 261 orthologs) was retained by selection of the sgRNA with the highest average efficiency (Supplementary Dataset 1a).

To further confirm that the designed sgRNAs target genes in the genome that are eventually transcribed, the flanking sequence of each sgRNA was BLASTed against the transcriptome of type A1 (*S. microadriaticum*) obtained from Baumgarten et al. (2013). Out of the 261 flanking sequences, 258 found a BLAST hit in the transcriptome (e-value $< 10^{-5}$, percent identity = 100%). Furthermore, the flanking sequences were BLASTed against the genome of type A1 and 221 of the 261 sgRNAs were found to reside on different scaffolds, indicating that the designed sgRNAs are likely to target different areas of the genome.

Detection of significantly enriched Gene Ontology (GO) terms associated with the 261 orthologs was performed with topGO package (version 2.26.0) in R using Fisher's exact test and the "weight01" algorithm implemented in the package (Alexa and Rahnenfuhrer, 2010). Significant Gene Ontology terms ($p < 0.05$) were identified (Supplementary Tables 2-4) and visualized using REVIGO (Supek et al., 2011) (Supplementary Figure 1).

sgRNA design for promoter studies

sgRNAs were designed to target upstream of the genes “Loci_144” and “Loci_1768” that were highly expressed across experimental conditions in the type A1 transcriptome (Baumgarten et al., 2013). The 1 kb sequence immediately preceding the start codon of each gene was extracted from the type A1 genome scaffolds 710 and 484 (Aranda et al., 2016) (matching Loci_144 and Loci_1768 in the type A1 transcriptome, respectively) (Baumgarten et al., 2013). These sequences were confirmed to contain all known *Symbiodinium* promoter elements (Lin et al., 2015), and the ends of the putative promoter regions were predicted using the method of Umarov and Solovyev (2017). The sgRNA with the highest efficiency and score located downstream from the potential splice leader acceptor (predicted promoter end) for each sequence was selected from a set of sgRNAs obtained by CRISPRseek (Zhu et al., 2014) (Supplementary Dataset 1b).

SUPPLEMENTARY TABLES AND FIGURE

Supplementary Table 1. Efficacy of G418 antibiotic capacity across diverse *Symbiodinium* variants. All *Symbiodinium* variants were cultured in the presence of 200 µg/ml kanamycin to remove bacteria. This ensured that the effects of G418 on *Symbiodinium* growth were not due to changes in the *Symbiodinium*-associated bacterial communities. *Symbiodinium* variants were exposed to four weeks of G418 treatment, with fresh G418 added biweekly. Growth at each G418 concentration was visually scored from - (no growth) up to +++ (growth equal to control).

Culture identity	ITS2 type	0 mg/ml G418	1.25 mg/ml G418	2.5 mg/ml G418	5 mg/ml G418	10 mg/ml G418
CCMP2464, rt61	A1	+++	-	-	-	-
CS159	A3	+++	+	-	-	-
CCMP2465, rt292	A3	+++	-	-	-	-
CS73	A3c	+++	+	-	-	-
CCMP2456, rt379	A4	+++	-	-	-	-
CCMP2469, JS879, rt80	A13	+++	+++	+	+	+
CCMP3345, CCMP2460, rt2	B1	+++	++	-	-	-
rt12	B1	+++	++	++	++	++
UTSB	B1	+++	+	-	-	-
CCMP3364	B2	+++	++	+	+	+
HHIB	C2	+++	++	-	-	-
rt203	C2	+++	++	-	-	-
PHMS T54 D1b	D1–5	+++	+	+	-	-
rt401	DS1	+++	++	+	-	-
UTSD	D1	+++	+++	+	+	+
CS156	F1	+++	+++	-	-	-
UTSC	F1	+++	++	+	+	+
CCMP2455, rt133	F2	+++	++	+	+	+

Supplementary Table 2. Significantly enriched (Fisher's exact test, $P < 0.05$) biological process gene ontology categories for which there are single copy orthologs with optimal CRISPR/Cas9 target sites conserved across all three *Symbiodinium* genomes.

GO.ID	Term	Annotated	Significant	Expected	topgoFisher
GO:0006265	DNA topological change	38	3	0.37	0.0058
GO:0097040	phthiocerol biosynthetic process	13	2	0.13	0.0068
GO:0097041	phenolic phthiocerol biosynthetic proces...	13	2	0.13	0.0068

GO:0000105	histidine biosynthetic process	13	2	0.13	0.0068
GO:0055114	oxidation-reduction process	1933	32	18.68	0.0072
GO:0016441	posttranscriptional gene silencing	61	3	0.59	0.0096
GO:0009410	response to xenobiotic stimulus	23	2	0.22	0.0096
GO:0035964	COPI-coated vesicle budding	1	1	0.01	0.0097
GO:0051684	maintenance of Golgi location	1	1	0.01	0.0097
GO:0090399	replicative senescence	1	1	0.01	0.0097
GO:0006007	glucose catabolic process	136	5	1.31	0.0104
GO:0006511	ubiquitin-dependent protein catabolic pr...	402	5	3.89	0.0113
GO:0010224	response to UV-B	51	3	0.49	0.0132
GO:0006450	regulation of translational fidelity	21	2	0.2	0.0173
GO:0071770	DIM/DIP cell wall layer assembly	22	2	0.21	0.0189
GO:0006707	cholesterol catabolic process	2	1	0.02	0.0192
GO:0070980	biphenyl catabolic process	2	1	0.02	0.0192
GO:0010497	plasmodesmata-mediated intercellular tra...	2	1	0.02	0.0192
GO:0042777	plasma membrane ATP synthesis coupled pr...	2	1	0.02	0.0192
GO:0052696	flavonoid glucuronidation	2	1	0.02	0.0192
GO:0052697	xenobiotic glucuronidation	2	1	0.02	0.0192
GO:0042351	'de novo' GDP-L-fucose biosynthetic proc...	2	1	0.02	0.0192
GO:0051661	maintenance of centrosome location	2	1	0.02	0.0192
GO:0007080	mitotic metaphase plate congression	2	1	0.02	0.0192
GO:0032929	negative regulation of superoxide anion ...	2	1	0.02	0.0192
GO:0072428	signal transduction involved in intra-S ...	2	1	0.02	0.0192
GO:0030157	pancreatic juice secretion	2	1	0.02	0.0192
GO:0032691	negative regulation of interleukin-1 bet...	2	1	0.02	0.0192
GO:0006975	DNA damage induced protein phosphorylati...	2	1	0.02	0.0192
GO:0045019	negative regulation of nitric oxide bios...	2	1	0.02	0.0192
GO:0044419	interspecies interaction between organis...	284	2	2.74	0.0194
GO:0046898	response to cycloheximide	3	1	0.03	0.0287
GO:0019543	propionate catabolic process	3	1	0.03	0.0287
GO:0097067	cellular response to thyroid hormone sti...	3	1	0.03	0.0287
GO:0006571	tyrosine biosynthetic process	3	1	0.03	0.0287
GO:0031100	organ regeneration	3	1	0.03	0.0287
GO:0006429	leucyl-tRNA aminoacylation	3	1	0.03	0.0287
GO:0032720	negative regulation of tumor necrosis fa...	3	1	0.03	0.0287

GO:0032728	positive regulation of interferon-beta p...	3	1	0.03	0.0287
GO:0048768	root hair cell tip growth	3	1	0.03	0.0287
GO:0009094	L-phenylalanine biosynthetic process	3	1	0.03	0.0287
GO:0071361	cellular response to ethanol	3	1	0.03	0.0287
GO:1901029	negative regulation of mitochondrial out...	3	1	0.03	0.0287
GO:0032695	negative regulation of interleukin-12 pr...	3	1	0.03	0.0287
GO:0034205	beta-amyloid formation	3	1	0.03	0.0287
GO:0009853	photorespiration	29	2	0.28	0.0318
GO:1901016	regulation of potassium ion transmembran...	75	2	0.72	0.038
GO:0010125	mycothiol biosynthetic process	4	1	0.04	0.0381
GO:0071918	urea transmembrane transport	4	1	0.04	0.0381
GO:0006122	mitochondrial electron transport, ubiqui...	4	1	0.04	0.0381
GO:0009102	biotin biosynthetic process	4	1	0.04	0.0381
GO:0032544	plastid translation	4	1	0.04	0.0381
GO:0006953	acute-phase response	4	1	0.04	0.0381
GO:0007059	chromosome segregation	155	3	1.5	0.0382
GO:1902600	hydrogen ion transmembrane transport	122	4	1.18	0.0383
GO:0046854	phosphatidylinositol phosphorylation	77	3	0.74	0.0386
GO:0006397	mRNA processing	353	9	3.41	0.043
GO:0033962	cytoplasmic mRNA processing body assembl...	5	1	0.05	0.0474
GO:0051552	flavone metabolic process	5	1	0.05	0.0474
GO:0043278	response to morphine	5	1	0.05	0.0474

Supplementary Table 3. Significantly enriched (Fisher's exact test, $P < 0.05$) molecular function gene ontology categories for which there are single copy orthologs with optimal CRISPR/Cas9 target sites conserved across all three *Symbiodinium* genomes.

GO.ID	Term	Annotated	Significant	Expected	topgoFisher
GO:0004298	threonine-type endopeptidase activity	13	3	0.12	0.00022
GO:0003917	DNA topoisomerase type I activity	16	3	0.15	0.00043
GO:0050897	cobalt ion binding	6	2	0.06	0.00131
GO:0004129	cytochrome-c oxidase activity	8	2	0.08	0.00242
GO:0008266	poly(U) RNA binding	1	1	0.01	0.0095
GO:0004510	tryptophan 5-monooxygenase activity	1	1	0.01	0.0095
GO:0008525	phosphatidylcholine transporter activity	1	1	0.01	0.0095
GO:0050577	GDP-L-fucose synthase activity	1	1	0.01	0.0095

GO:0004505	phenylalanine 4-monooxygenase activity	1	1	0.01	0.0095
GO:0071208	histone pre-mRNA DCP binding	1	1	0.01	0.0095
GO:0034617	tetrahydrobiopterin binding	1	1	0.01	0.0095
GO:0004664	prephenate dehydratase activity	1	1	0.01	0.0095
GO:0070463	tubulin-dependent ATPase activity	1	1	0.01	0.0095
GO:0051959	dynein light intermediate chain binding	1	1	0.01	0.0095
GO:0008767	UDP-galactopyranose mutase activity	1	1	0.01	0.0095
GO:0042286	glutamate-1-semialdehyde 2,1-aminomutase...	1	1	0.01	0.0095
GO:0047547	2-methylcitrate dehydratase activity	1	1	0.01	0.0095
GO:0000293	ferric-chelate reductase activity	1	1	0.01	0.0095
GO:0008710	8-amino-7-oxononanoate synthase activity	1	1	0.01	0.0095
GO:0004399	histidinol dehydrogenase activity	1	1	0.01	0.0095
GO:0005244	voltage-gated ion channel activity	544	6	5.17	0.01393
GO:0002161	aminoacyl-tRNA editing activity	21	2	0.2	0.01673
GO:0004046	aminoacylase activity	2	1	0.02	0.0189
GO:0004783	sulfite reductase (NADPH) activity	2	1	0.02	0.0189
GO:0004771	sterol esterase activity	2	1	0.02	0.0189
GO:0004560	alpha-L-fucosidase activity	2	1	0.02	0.0189
GO:0000287	magnesium ion binding	288	7	2.74	0.02041
GO:0047830	D-octopine dehydrogenase activity	3	1	0.03	0.02822
GO:0047617	acyl-CoA hydrolase activity	3	1	0.03	0.02822
GO:0004823	leucine-tRNA ligase activity	3	1	0.03	0.02822
GO:0047456	2-methylisocitrate dehydratase activity	3	1	0.03	0.02822
GO:0001972	retinoic acid binding	3	1	0.03	0.02822
GO:0004834	tryptophan synthase activity	3	1	0.03	0.02822
GO:0043394	proteoglycan binding	3	1	0.03	0.02822
GO:0005506	iron ion binding	355	8	3.37	0.03207
GO:0003756	protein disulfide isomerase activity	31	2	0.29	0.03484
GO:0008526	phosphatidylinositol transporter activit...	4	1	0.04	0.03745
GO:0008986	pyruvate, water dikinase activity	4	1	0.04	0.03745
GO:0003796	lysozyme activity	4	1	0.04	0.03745
GO:0015204	urea transmembrane transporter activity	4	1	0.04	0.03745
GO:0004649	poly(ADP-ribose) glycohydrolase activity	4	1	0.04	0.03745
GO:0031210	phosphatidylcholine binding	4	1	0.04	0.03745
GO:0003727	single-stranded RNA binding	36	3	0.34	0.04309
GO:0004315	3-oxoacyl-[acyl-carrier-protein] synthas...	36	2	0.34	0.04577
GO:0008705	methionine synthase activity	5	1	0.05	0.04659

GO:0004477	methenyltetrahydrofolate cyclohydrolase ...	5	1	0.05	0.04659
GO:0004605	phosphatidate cytidylyltransferase activ...	5	1	0.05	0.04659

Supplementary Table 4. Significantly enriched (Fisher's exact test, P < 0.05) cellular compoent gene ontology categories for which there are single copy orthologs with optimal CRISPR/Cas9 target sites conserved across all three *Symbiodinium* genomes.

GO.ID	Term	Annotated	Significant	Expected	topgoFisher
GO:0048046	apoplast	51	5	0.57	0.00026
GO:0019773	proteasome core complex, alpha-subunit c...	6	2	0.07	0.00181
GO:0030140	trans-Golgi network transport vesicle	13	2	0.15	0.00895
GO:0034081	polyketide synthase complex	14	2	0.16	0.01036
GO:0002102	podosome	1	1	0.01	0.01119
GO:0070765	gamma-secretase complex	1	1	0.01	0.01119
GO:0042589	zymogen granule membrane	1	1	0.01	0.01119
GO:0034715	pICln-Sm protein complex	1	1	0.01	0.01119
GO:0042717	plasma membrane-derived chromatophore me...	1	1	0.01	0.01119
GO:0009941	chloroplast envelope	207	6	2.32	0.01556
GO:0009535	chloroplast thylakoid membrane	240	7	2.69	0.01842
GO:0022626	cytosolic ribosome	107	4	1.2	0.01855
GO:0070069	cytochrome complex	8	2	0.09	0.02212
GO:0036021	endolysosome lumen	2	1	0.02	0.02225
GO:0034719	SMN-Sm protein complex	2	1	0.02	0.02225
GO:0009337	sulfite reductase complex (NADPH)	2	1	0.02	0.02225
GO:0034709	methylosome	2	1	0.02	0.02225
GO:0030017	sarcomere	274	2	3.07	0.02254
GO:0016605	PML body	21	2	0.23	0.02273
GO:0009570	chloroplast stroma	185	6	2.07	0.02568
GO:0005730	nucleolus	381	9	4.26	0.0276
GO:0009534	chloroplast thylakoid	264	9	2.95	0.02776
GO:0005828	kinetochore microtubule	3	1	0.03	0.03319
GO:0042175	nuclear outer membrane-endoplasmic retic…	484	5	5.42	0.03349
GO:0042470	melanosome	26	2	0.29	0.03392
GO:0005687	U4 snRNP	4	1	0.04	0.04401
GO:0071339	MLL1 complex	4	1	0.04	0.04401
GO:0097362	MCM8-MCM9 complex	4	1	0.04	0.04401

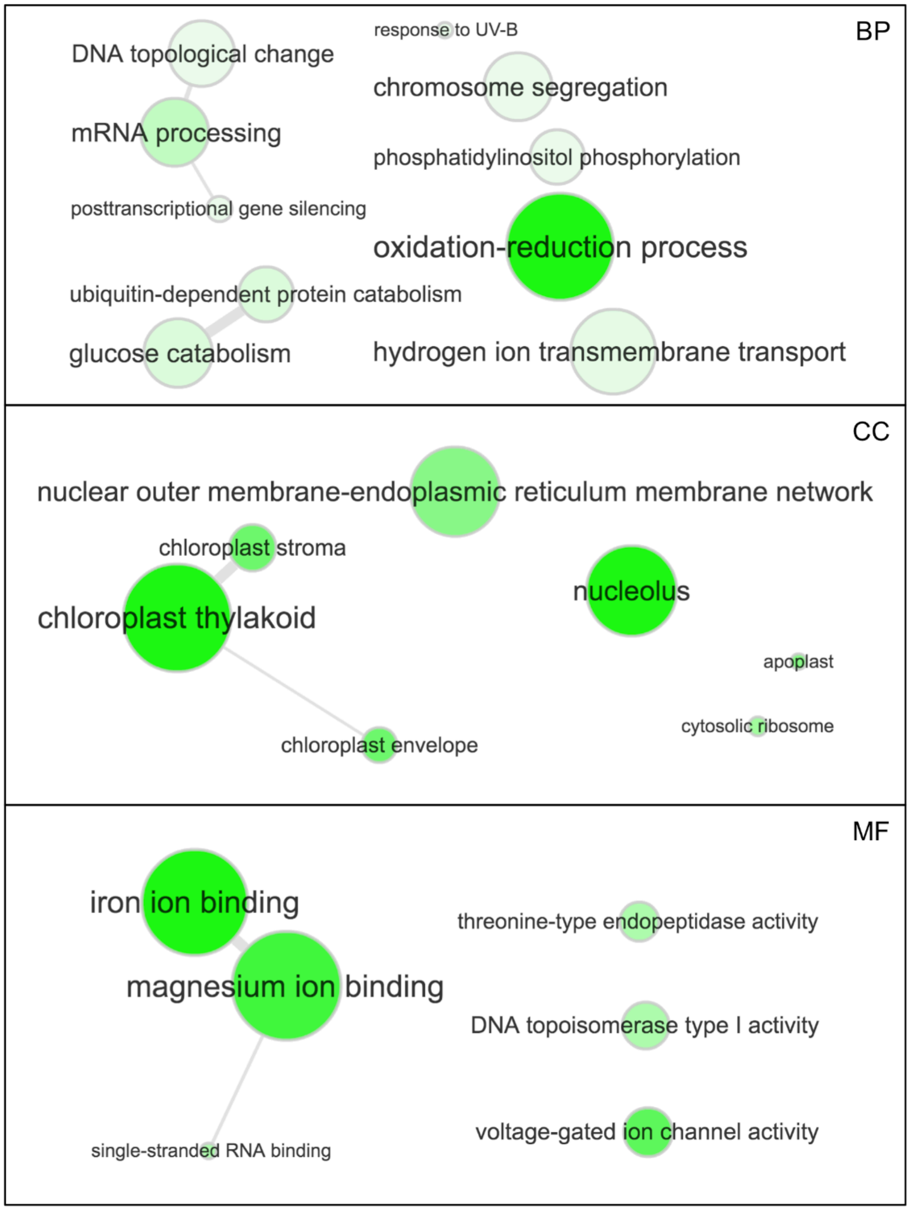

Supplementary Figure 1
Functional gene groups for CRISPR/Cas9 genome editing of *Symbiodinium*.
Significantly enriched gene ontology (GO) categories (Fisher's exact test, $P < 0.05$) for which there are at least three single copy orthologs with optimal CRISPR/Cas9 target sites conserved across all three *Symbiodinium* genomes. Relationship graphs were generated separately for biological process (BP), cellular component (CC), and molecular function (MF) GO categories using REVIGO. Redundant categories (similarity > 0.9) were collapsed into the most common equivalent category in the UniProt database. Bubble size indicates the relative frequency of the category in the UniProt database. Darker bubble color denotes categories that contain more single copy orthologs with conserved CRISPR/Cas9 target sites. The width of the lines connecting categories represents the similarity between categories.

SUPPLEMENTARY REFERENCES

Alexa, A., and Rahnenfuhrer, J. (2010). topGO: enrichment analysis for gene ontology. *R package version* 2(0).

Aranda, M., Li, Y., Liew, Y., Baumgarten, S., Simakov, O., Wilson, M., et al. (2016). Genomes of coral dinoflagellate symbionts highlight evolutionary adaptations conducive to a symbiotic lifestyle. *Sci. Rep.* 6.

Baumgarten, S., Bayer, T., Aranda, M., Liew, Y.J., Carr, A., Micklem, G., et al. (2013). Integrating microRNA and mRNA expression profiling in *Symbiodinium microadriaticum*, a dinoflagellate symbiont of reef-building corals. *BMC Genomics* 14.

Lechner, M., Findeiß, S., Steiner, L., Marz, M., Stadler, P.F., and Prohaska, S.J. (2011). Proteinortho: detection of (co-)orthologs in large-scale analysis. *BMC Bioinformatics* 12.

Lin, S., Cheng, S., Song, B., Zhong, X., Lin, X., Li, W., et al. (2015). The *Symbiodinium kawagutii* genome illuminates dinoflagellate gene expression and coral symbiosis. *Science* 350**,** 691-694.

Shoguchi, E., Shinzato, C., Kawashima, T., Gyoja, F., Mungpakdee, S., Koyanagi, R., et al. (2013). Draft assembly of the *Symbiodinium minutum* nuclear genome reveals dinoflagellate gene structure. *Curr. Biol.* 23**,** 1399-1408.

Supek, F., Bošnjak, M., Škunca, N., and Šmuc, T. (2011). REVIGO summarizes and visualizes long lists of gene ontology terms. *PloS One* 6**,** e21800.

Umarov, R.K., and Solovyev, V.V. (2017). Recognition of prokaryotic and eukaryotic promoters using convolutional deep learning neural networks. *PloS One* 12**,** e0171410.

Zhu, L.J., Holmes, B.R., Aronin, N., and Brodsky, M.H. (2014). CRISPRseek: a bioconductor package to identify target-specific guide RNAs for CRISPR-Cas9 genome-editing systems. *PLoS One* 9**,** e108424.
